# LOC100996425 acts as a promoter in prostate cancer by mediating hepatocyte nuclear factor 4A and the AMPK/mTOR pathway

**DOI:** 10.1111/jcmm.16657

**Published:** 2021-07-26

**Authors:** Xiuyan Wang, Bin Song, Mingcui Zang, He Ji, He Yang, Shuang Jiang, Xiao Yang

**Affiliations:** ^1^ Department of Urology The Second Hospital of Jilin University Changchun China; ^2^ Department of Neurosurgery The Second Hospital of Jilin University Changchun China; ^3^ Department of Hepatobiliary and Pancreatic Surgery I The First Hospital of Jilin University Changchun China; ^4^ Department of Gynaecology The Second Hospital of Jilin University Changchun China

**Keywords:** AMPK, hepatocyte nuclear factor 4A, LOC100996425, long non‐coding RNA, mTOR pathway, prostate cancer

## Abstract

The involvement of long non‐coding RNAs (lncRNAs), differentially expressed genes and signals in prostate cancer (PCa) continues to be a subject of investigation. This study determined effects of LOC100996425 on human PCa by targeting hepatocyte nuclear factor 4A (HNF4A) via the AMPK/mTOR pathway. PCa and adjacent normal tissues were obtained to characterize expression pattern of LOC100996425, HNF4A and the AMPK/mTOR pathway‐related genes. Then, the target gene of LOC100996425 was determined with lncRNA target prediction website and further verification was obtained through luciferase assay and ribonucleoprotein immunoprecipitation. After that, PCa cells were introduced with LOC100996425, HNF4A, siLOC100996425 or siHNF4A to explore the specific significance of LOC100996425 and HNF4A in PCa. The mechanism associated with AMPK/mTOR pathway was investigated using AMPK inhibitor or activator. LOC100996425 was up‐regulated, while HNF4A was down‐regulated in the PCa tissues. HNF4A was a target gene of LOC100996425. PCa cells transfected with either siLOC100996425 or HNF4A displayed reduced rates of PCa cell proliferation and migration while elevating cell apoptosis. HNF4A overexpression reversed the promotive effect of LOC100996425 overexpression on PCa. The activation of AMPK pathway involved in the cancer progression mediated by LOC100996425. Down‐regulation of LOC100996425 retards progression of PCa through HNF4A‐mediated AMPK/mTOR pathway.

## 1 INTRODUCTION

Prostate cancer (PCa) is an androgen‐dependent disease, which is responsive to treatment during its early stages but becomes resistant to existing therapeutic approaches at its later stages.^1^ There are a number of both hereditary and exogenous factors that have been identified in existing literature as primary risk factors in the development of PCa.^2^, ^3^ The clinical manifestations of newly diagnosed patients with metastatic PCa include bone pain, regression of soft‐tissue metastases, in addition to reduced levels of serum prostate‐specific antigen (PSA) which could potentially occur due to a rapid response to surgical or medical castration.^4^ The current established therapies for PCa possess the ability to eradicate a large number of cells within the tumour, and however, they do not provide a complete cure from the disease among the greater majority of diagnosed patients.^5^

LncRNAs represent a unique type of non‐coding RNAs which regulate tumour behaviour and various cellular processes.^6^, ^7^, ^8^ The aberrant expressions of lncRNAs implicated in various cancers, including breast cancer, colorectal cancer.^9^, ^10^ LncRNAs continue to be investigated due to their potential as emerging biomarkers that could well facilitate the delivery of more efficient diagnosis and prognosis for patients suffering from PCa.^11^ In the present study, we highlight potential impact of a novel lncRNA LOC100996425 on PCa, with a particular emphasis on the mechanisms and effects elicited by lncRNAs.

LncRNAs control mRNA stability and splicing, while also exhibiting a certain degree of control on the recruitment transcription factors.^12^, ^13^ Several studies have highlighted role of lncRNAs in various types of cancers may as well as linking them with hepatocyte nuclear factor 4A (HNF4A).^14^, ^15^ HNF4A is a transcription factor part of the nuclear hormone receptor superfamily, interacting with regulatory elements in promoters and enhancers of genes associated with cholesterol, fatty acids and glucose metabolism.^16^ HNF4A possesses the ability to regulate the expression of multiple components within three major compartments including desmosomes, adherens and tight junction.^17^ Moreover, HNF4A was also reported to be a potential biomarker in several cancers, such as breast cancer, liver cancer and colon cancer.^18^, ^19^, ^20^ However, the correlation between HNF4A and PCa was still to be investigated. Adenosine 5’‐monophosphate‐activated protein kinase (AMPK), is a ubiquitous serine/threonine protein kinase and regulates cellular energy metabolism.^21^ The mammalian target of rapamycin (mTOR) is a cell growth regulator which unifies growth factor and nutrient signals.^22^ The AMPK/mTOR pathway has been reported to regulate autophagy to react to numerous anticancer agents.^23^ As HNF4A predicted to be targeted by LOC100996425 from the website lncRNA targets, it is speculated that LOC100996425 might function in PCa by targeting HNF4A. Thus, we investigated role by which LOC100996425 influences malignancy in PCa by targeting HNF4A *via* AMPK/mTOR pathway.

## 2 METHODS

### 2.1 Ethics statement

This study was conducted with the approval of the Ethics Committee of The Second Hospital of Jilin University. All participants signed written informed consent documentation prior to enrolment.

### 2.2 Study subjects

A total of 110 PCa tissue samples (the PCa group) were obtained from patients previously undergone surgical resection procedures at The Second Hospital of Jilin University between January 2011 and September 2015. The corresponding adjacent normal tissue samples served as the control group. All patients who did not receive radiotherapy, chemotherapy or immunotherapy prior to surgery were included. Patients were diagnosed with PCa by magnetic resonance spectroscopy (MRS). The average age was 65.33 ± 10.35 years (range: 37‐78 years). According to the Gleason score system, there were 35 Gleason I cases (well‐differentiated PCa, less nuclear atypia), 30 Gleason II cases (well‐differentiated PCa with some nuclear atypia), 45 cases of Gleason III (moderately differentiated PCa with some nuclear atypia).^24^

### 2.3 Haematoxylin‐eosin staining

Portions of the PCa and adjacent normal tissues were immersed in 4% paraformaldehyde for 12 hours, dehydrated, cleared in xylene (YB‐8499, Shanghai Yu Bo Biological Technology, Shanghai, China), embedded with paraffin and sectioned (4 μm). After dewaxing twice in xylene (5 min/time), the tissue sections were dehydrated, stained with haematoxylin for 2 minutes, differentiated in 1% hydrochloric acid alcohol for 10 seconds and stained with eosin for 1 minutes. The sections were subsequently dehydrated, cleared and mounted. The PCa and adjacent normal tissues were then observed under a microscope and photographed.

### 2.4 Immunohistochemistry

The paraffin‐embedded sections were dried, dewaxed with xylene, dehydrated and immersed in PBS. Following high‐pressure antigen retrieval using citrate buffer (0.01 mol/L) for 10 minutes and endogenous peroxidase activity blockade with 0.3% H_2_O_2_‐methanol for 20 minutes, sections were incubated with 10% goat serum (36119ES03, Shanghai Yeasen Biotechnology Co., Ltd) for 10 minutes, with rabbit anti‐human HNF4A antibody (1:2000 dilution, ab181604, Abcam Inc) at 37°C for 1 hour, which was subsequently replaced by PBS as a negative control (NC). The sections were then incubated with the NC overnight at 4°C. Later, horseradish peroxidase (HRP)‐conjugated goat anti‐rabbit immunoglobulin G (IgG) secondary antibody (1:1000, ab6721, Abcam Inc) was added for incubation purposes over a period of 30 minutes. Sections subsequently stained with diaminobenzidine (DAB, P0203, Beyotime) for 5 minutes with a microscope employed to regulate the degree of staining. After 3 washes under running water (5 min/time), sections were counterstained with haematoxylin for 3 minutes, differentiated, returned to blue, mounted, observed under an optical microscope (XDS‐800D, Shanghai Caikon Optical Instrument Co., Ltd.) and photographed accordingly. Positive expression rate was then determined based on the selection of five randomly selected fields from each section. The average absorbance of each field was calculated using Image‐Pro Plus Version 7.0.

### 2.5 Plasmid construction

Based on the sequences of LOC100996425 and the HNF4A transcripts in GenBank, full‐length, siRNA and NC sequences of LOC100996425 and HNF4A were designed using the Ambion website (www.ambion.com) and then synthesized by the Shanghai GeneChem Co., Ltd. Each gene was designed with 3 pairs of siRNA sequences. The correct sequence was cloned into Hind III and Xho I restriction sites of the plasmid vector pcDNA3.1 (VPI0001, Invitrogen) at 16°C for 1 hour. Once the recombinant plasmids had been transferred into competent DH5α cells (D9052, Takara), the resistant colonies were subsequently screened out and identified by means of polymerase chain reaction (PCR), followed by positive clone sequencing. The plasmids were then promptly extracted in small quantities and stored at −20°C. The siRNA sequences are shown in Table S1.

### 2.6 Cell grouping and transfection

The human PCa cell lines C4‐2, PC‐3, 22RV1, LNCap and DU‐145 as well as normal prostate cell line WPMV‐1 (as NC) were purchased from Procell. All the cells were identified by short tandem repeat (STR). The cell lines of the samples were calculated by comparing the results of STR typing with the professional cell STR database. HNF4A expression in the cell lines was determined for cell line screening. The cells were passaged and seeded into 6‐well plates 24 hours. Cells reaching 30%‐50% confluence were transfected with NC empty vector, LOC100996425 plasmids, HNF4A plasmids, NC plasmids of siRNA, siRNA targeting LOC100996425 (siLOC100996425) and/or siHNF4A. Cell transfection was performed using Lipofectamine 2000. AMPK inhibitor (dorsomorphin, 10 µmol/L, APExBIO, CAS# 866405‐64‐3) and AMPK activator (A769662, 100 µmol/L, APExBIO, CAS# 844499‐71‐4) were applied to inhibit or activate the AMPK pathway. 100 pmol siRNA or 2 µg plasmids were diluted with 250 µL of serum‐free RPMI 1640 medium. Lipofectamine 2000 (5 µL) was diluted with 250 μL serum‐free RPMI 1640 medium. Next, the above two diluted plasmid solutions were mixed with Lipofectamine 2000, followed by a 20 minutes period of incubation, after which they were moved into the wells of a culture plate. Following transfection for 6‐8 hours, cells were cultured with complete medium for 24‐48 hours for subsequent experimentation.

### 2.7 Dual‐luciferase reporter gene assay

The binding sites of LOC100996425 and HNF4A were predicted using a Bioinformatics website Starbase (http://starbase.sysu.edu.cn/index.php). Dual‐luciferase reporter gene assay was performed to ascertain as to whether HNF4A was a direct target of LOC100996425. The artificially synthesized gene segment of HNF4A 3’‐untranslated region (3’UTR) was introduced into pMIR‐reporter (HUAYUEYANG Technology (Beijing) Co., Ltd.). The mutant (Mut) sequence was designed based on the wild‐type (Wt) HNF4A. Afterwards, the target segments were subsequently inserted into the pMIR‐reporter. The Wt and Mut plasmids with correct sequence were subsequently transfected with LOC100996425 into the human embryonic kidney (HEK)‐293T cells (Shanghai Beinuo Biotechnology). Luciferase activity was detected using a dual‐luciferase reporter gene assay kit (K801‐200, BioVision).

### 2.8 Ribonucleoprotein immunoprecipitation (RNP‐IP)

When cell confluence reached approximately 80%, the DU‐145 cells were digested by 0.25% trypsin and centrifuged. The cell precipitation was lysed with polyribosome lysate, followed by addition of 1 mmol/L β‐mercaptoethanol and 100 U/mL ribonuclease inhibitor as protective agents (Roche Diagnostics GmbH), a mixture of Halt protease and phosphatase inhibitor (Thermo Fisher Scientific Inc) as well as 25 μmol/L MG 132 (Sigma). The lysed products were placed on ice for 30 minutes and centrifuged at 12 000 × *g* for 5 minutes followed by supernatant collection. The total RNA, which had previously been separated from 5% of the aforementioned lysed products, was employed as a control to compare the extracted RNA to the various samples. RPLP0 mRNA in RNA obtained from the lysed products was regarded as the internal reference. Following cell lysis, 2 μg of monoclonal antibody rat anti‐Ago‐2 (MABE253, Millipore) or IgG (sc‐2026, Santa Cruz Biotechnology, Inc) was added and incubated at 4°C overnight. The following day, 40 μL G protein‐coupled agarose beads (Santa Cruz Biotechnology) were added to the lysate, followed by incubation at 4°C overnight under continual shaking conditions. Next, the pre‐cooled NT2 buffer was employed to clean the agarose beads twice. After cleaned, the immunoprecipitates were transferred into the NT2 buffer containing 100 g/mL protease K (Sigma) and permitted to react at 55°C for 30 minutes. After reaction, AccuZol RNA was extracted and directly added to the immunoprecipitates. The RNA enriched in the Ago2 complex was then separated with further analysis was subsequently performed by RT‐qPCR.

### 2.9 RT‐qPCR

Total RNA was extracted from both the PCa and adjacent normal tissues. Reverse transcription was performed using the reverse transcription kit (K1621, Fermentas). The primers (Table S2) were designed and synthesized by Shanghai GeneChem Co., Ltd. Gene expressions were detected using a fluorescent qPCR kit (Takara). RT‐qPCR was performed with a PCR kit (ABI 7500, ABI). GAPDH was considered as the internal reference. The 2^‐ΔΔCt^ method was applied to calculate mRNA expression.

### 2.10 Western blot analysis

50 mg tissue samples were lysed with protein lysate (R0010; Beijing Solarbio Science & Technology Co., Ltd.) and centrifuged with supernatant collected accordingly. The sampling wells (20 μg/well) were then added to the pre‐treated protein for protein isolation purposes on 10% SDS‐PAGE (P1200, Solarbio). Electrophoresis was initially performed at 8 V/cm, which was turned up to 15 V/cm once the protein had been transferred into the separation gel. The protein samples were subsequently transferred onto polyvinylidene fluoride membranes (HVLP04700, Millipore), which were blocked with 5% skimmed milk for 2 hours, and incubated in a 4°C refrigerator overnight with diluted rabbit anti‐human HNF4A (1:1000, ab181604), AMPK (1:2000, ab32047), mTOR (1:2000, ab32028), LC3‐II (1:2000, ab192890), Beclin‐1 (1:2000, ab207612), Bcl‐2 (1:2000, ab32124), Bax (1:2000, ab32503), phosphorylated (p)‐AMPK (1:2000, ab68206), p‐mTOR (1:2000, ab84400), PCNA (1:2000, ab92552) and β‐actin (1:1000, ab8227) antibody, which were all purchased from Abcam Inc. The samples were incubated with horseradish peroxidase (HRP)‐conjugated goat anti‐rabbit IgG antibody (1:2000, ab6721, Abcam Inc) for 2 hours. DAB was applied for coloration purposes, while the Gel Doc XR imager system (Bio‐Rad Laboratories, Inc CA) was employed for images analyses.

### 2.11 MTT assay

The PCa cells were seeded into a 96‐well plate at 1 × 10^4^ cells/well. After 8 parallel wells were set, the culture medium was added into the wells without cells, which were used as the blank control. When cell confluence had reached 70%, each well was added with 10 μL MTT solution (5 mg/mL, ST316, Beyotime) and incubated at 37°C for 4 hours. Cells were added with 100 μL dimethyl sulfoxide (DMSO, D5879, Sigma). OD value was then measured using a microplate reader (MK3, Thermo Fisher Scientific Inc).

### 2.12 Scratch test

After a 48‐hour period of transfection, the cells were inoculated into a 6‐well plate. When reached 90%‐100% confluence, the cell monolayers were wounded by means of scratching. The cells were then rinsed with PBS for debris removal purposes, followed by incubation in a cell incubator. Images of the wounds were acquired at 0 hour and 24 hours time‐points. Cell migration distance was then observed and measured.

### 2.13 Flow cytometry

The cells were treated with 0.25% trypsin and centrifuged at 201 × *g* at 4°C for 5 minutes twice. Pre‐cooled 70% ethanol was added in order to fix the cells at 4°C overnight. After centrifugation at 201 × *g* at 4°C for 5 minutes and removal of the supernatant, cells were centrifuged again, added with 10 μL RNase enzyme and stained with 1% propidium iodide (PI, 40710ES03, Shanghai qcbio Science & Technologies co., Ltd, Shanghai, China) for 30 min in the dark condition. Red fluorescence was then collected at 488 nm using a FACSCalibur flow cytometer (BD Biosciences) to detect cell cycle.

After a 48‐hour period transfection, cells were detached with EDTA‐free trypsin, collected, and centrifuged at 4°C at 201 × *g* for 5 minutes. Once the supernatant had been removed, the cells were centrifuged again, cell apoptosis was detected using an Annexin‐V‐FITC/PI apoptosis detection kit (CA1020, Solarbio). Cell apoptosis was analysed with the use of a flow cytometer.

### 2.14 Follow‐up

Follow‐up was conducted in 110 PCa patients, with December 2017 set as the deadline. The patients were followed up through telephone or return visit to the hospital to determine the overall survival which was defined as the time from random assignment to death as a result of any given cause. The 3‐year overall survival of patients in each group was then determined. At the end of the follow‐up, 21 patients out of 110 patients were lost to follow‐up, with the follow‐up rate determined to be 89.09%. The follow‐up period was 5 to 36 months.

### 2.15 Statistical analysis

Data were expressed as mean ± standard deviation. A paired *t* test was employed for comparison between two groups, while an unpaired *t* test for comparisons among multiple groups. Comparisons among multiple groups were assessed using one‐way analysis of variance (ANOVA), followed by Tukey's post hoc test. Two‐way ANOVA was used to compare the data among groups at different time‐points, followed by Bonferroni post hoc test. Kaplan‐Meier survival curve was constructed in order to calculate the patient's overall survival rate, with overall survival rate differences analysed using a log‐rank test. *P* < .05 was of statistical significance.

## 3 RESULTS

### 3.1 Low expression of HNF4A is detected in the PCa tissues

**FIGURE 1:**
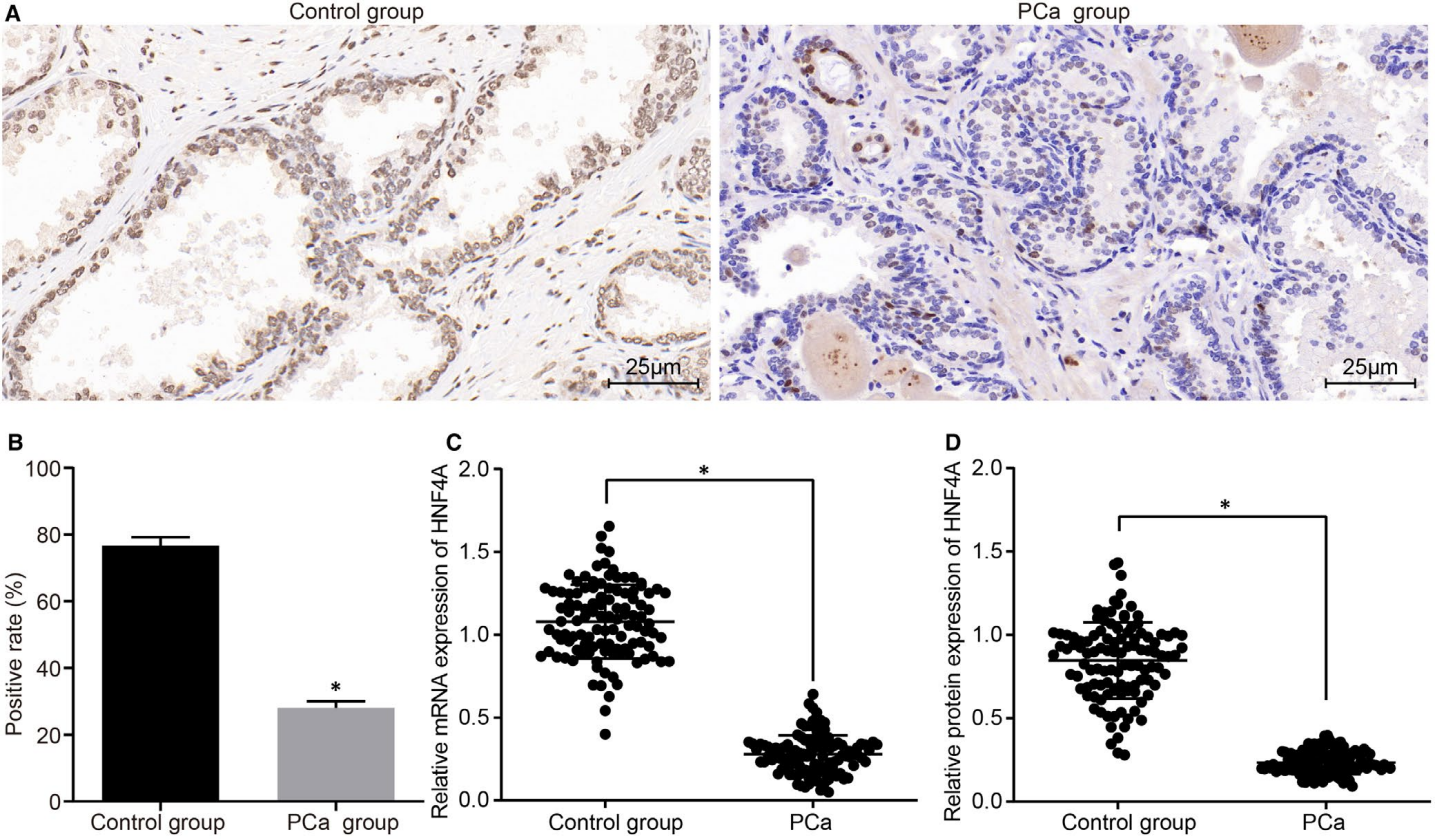
PCa tissues present lower expression of HNF4A. A, staining images of positive expression of HNF4A protein in PCa and corresponding adjacent normal tissues of PCa patients, observed after immunohistochemistry (×400) (6 patients were randomly selected for detection. Figure 1A shows a group of representative experimental results). B, comparison of positive expression rate of HNF4A between the PCa and adjacent normal tissues based on immunohistochemistry. C, HNF4A mRNA expression in PCa and corresponding adjacent normal tissues obtained from the 110 PCa patients. D, HNF4A protein expression in PCa and corresponding adjacent normal tissues obtained from the 110 PCa patients. The data were expressed as mean ± standard deviation, and analysed by the paired *t* test. PCa, prostate cancer; HNF4A, hepatocyte nuclear factor 4A. *P < .05 vs. the control

The positive expression of HNF4A was predominately observed in the nucleus, with the positive granules considered to be reflected by brown (Figure 1A; Figure S1). The positive rate of HNF4A in the adjacent normal tissues was (76.7 ± 2.5)%, which was higher than PCa tissues (28.0 ± 2.0)% (Figure 1B). mRNA and protein expression of HNF4A in PCa tissues was y lower than adjacent normal tissues (Figure 1C, D; Table S3; Figure S1).

### 3.2 LOC100996425 expression is increased in PCa tissues and correlated with a lower overall survival rate of PCa patients

**FIGURE 2:**
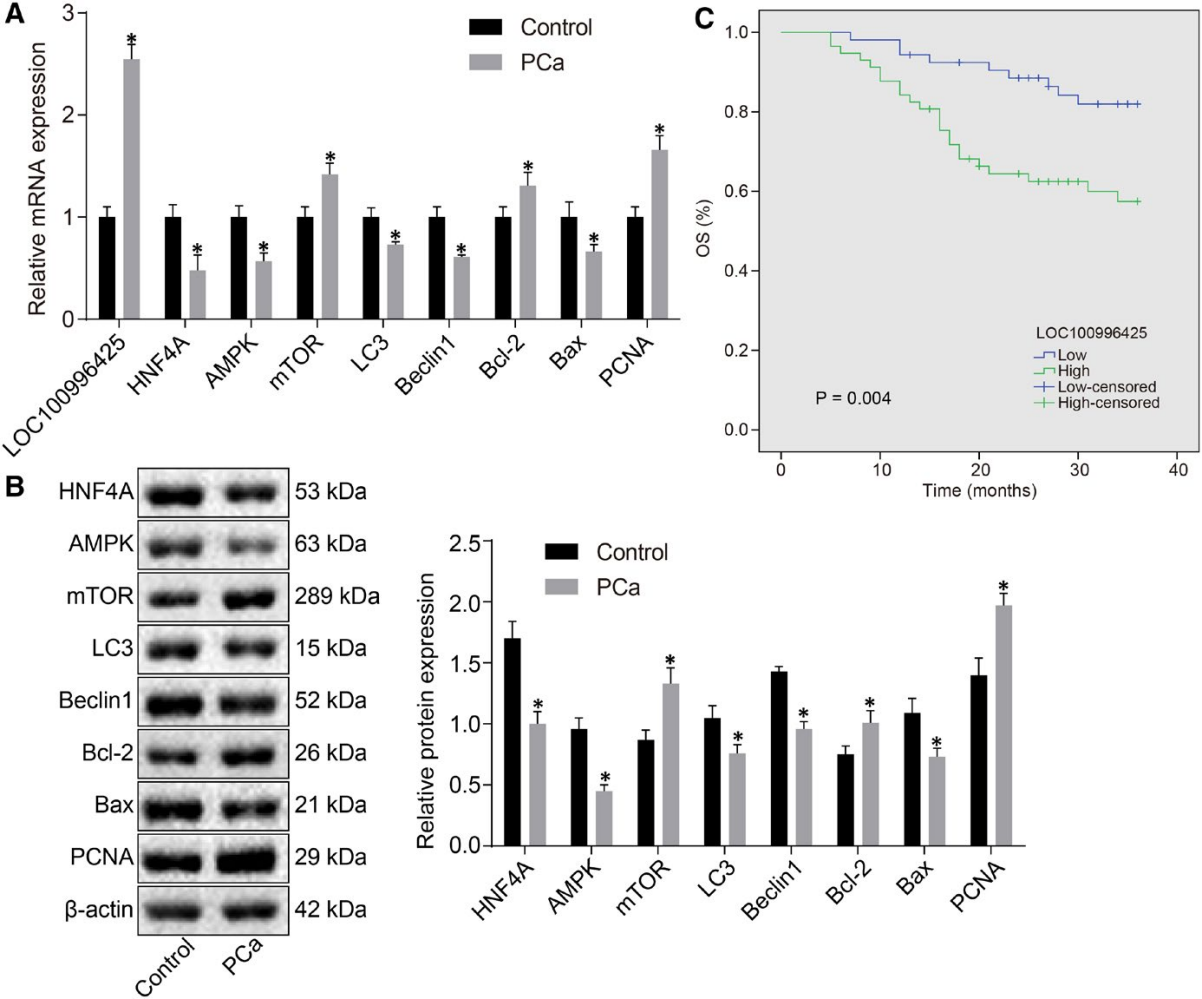
LOC100996425 is up‐regulated in PCa tissues and correlated with lower overall survival rate of PCa patients. A, mRNA expression of mTOR, Bcl‐2, and PCNA, HNF4A, AMPK, LC3, Beclin‐1 and Bax in PCa and adjacent normal tissues, as detected by RT‐qPCR. B, protein expression of mTOR, Bcl‐2, and PCNA, HNF4A, AMPK, LC3, Beclin‐1, and Bax in PCa and adjacent normal tissues measured by Western blot analysis. C, the correlation between the LOC100996425 expression and overall survival rate of PCa patients analysed through Kaplan‐Meier survival curve analysis; the data were expressed as mean ± standard deviation and analysed by the paired *t* test. AMPK, adenosine 5’‐monophosphate‐activated protein kinase; mTOR, mammalian target of rapamycin; Bcl‐2, B cell lymphoma‐2; LC3, light chain 3; PCNA, proliferating cell nuclear antigen; RT‐qPCR, reverse transcription quantitative polymerase chain reaction. *P < .05 vs. the control

LOC100996425 expression (Figure 2A) and expression of mTOR, Bcl‐2 and PCNA (Figure 2A, B) were higher in the PCa tissues, while mRNA and protein expressions of HNF4A, AMPK, LC3, Beclin‐1 and Bax (Figure 2A, B) were reduced in comparison with adjacent normal tissues. Additionally, patients were assigned into the highly expressed LOC100996425 (n = 57) and the poorly expressed LOC100996425 (n = 53) group. The overall survival rate was determined to be markedly higher among patients with poorly expressed LOC100996425 than those with highly expressed LOC100996425 (*P* = .004), in accordance with the findings from the Kaplan‐Meier survival curves and analysis of log‐rank test (Figure 2C).

### 3.3 Cell line selection and transfection of siRNA and overexpression plasmids

**FIGURE 3:**
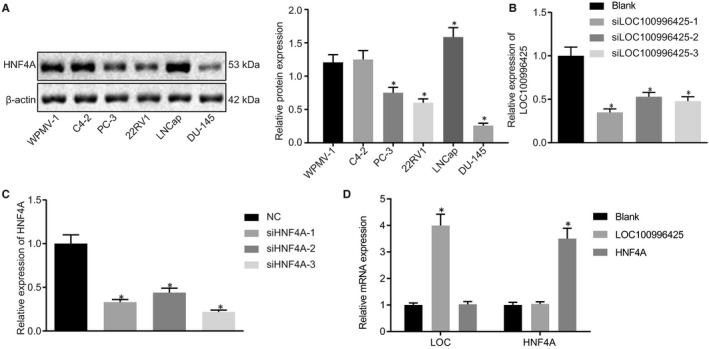
Cell line selection and transfection of siRNA and overexpression plasmids. A, protein expression of HNF4A in PCa cell lines C4‐2, PC‐3, 22RV1, LNCap and DU‐145, and normal prostate cell line WPMV‐1, measured by Western blot analysis, the results were obtained from three independent experiments. B, interference effect of siLOC100996425. C, interference effect of siHNF4A. D, transfection efficiency of siRNA and overexpression plasmids. The data were expressed as mean ± standard deviation and analysed by the one‐way ANOVA, followed by Tukey's post hoc test. siRNA, small interfering RNA. *P < .05 vs. the blank or NC plasmid‐transfected cells

Among the PCa cell lines C4‐2, PC‐3, 22RV1, LNCap and DU‐145, HNF4A had the lowest expression in DU‐145 cells. Therefore, the DU‐145 cells were selected for subsequent experimentation (Figure 3A, B). The siLOC100996425‐1 and siHNF4A‐3 presented with a relatively high interference effect (Figure 3B‐D). In DU‐145 cells, both LOC100996425‐1 and HNF4A mRNA levels were increased after their overexpression.

### 3.4 HNF4A is a target of LOC100996425

**FIGURE 4:**
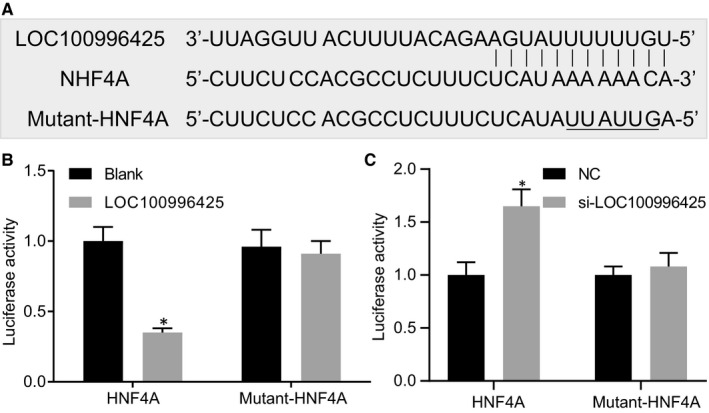
HNF4A is a target gene of LOC100996425. A, gene sequences of LOC100996425, HNF4A and mutant‐HNF4A. B and C, the relationship between LOC100996425 and HNF4A, verified by luciferase activity determination. The data were analysed by unpaired *t* test. *P < .05 *vs*. the blank or NC‐transfected cells

The results obtained from the Bioinformatics website RNA22 indicated that LOC100996425 contained a binding site with the mRNA sequence of HNF4A in its 3’‐UTR (Figure 4A). In contrast to the cells transfected with NC, the relative luciferase activity of HNF4A in the cells transfected with LOC100996425 vector was decreased (Figure 4B), while that in the cells transfected with siLOC100996425 cells was increased (Figure 4C). For further verification, we mutated the binding site and then carried out dual‐luciferase reporter gene assay again. No matter overexpression or silencing of LOC100996425, the luciferase activity of HNF4A was not significantly changed (Figure 4B, C), suggesting that HNF4A was negatively regulated by LOC100996425.

**FIGURE 5:**
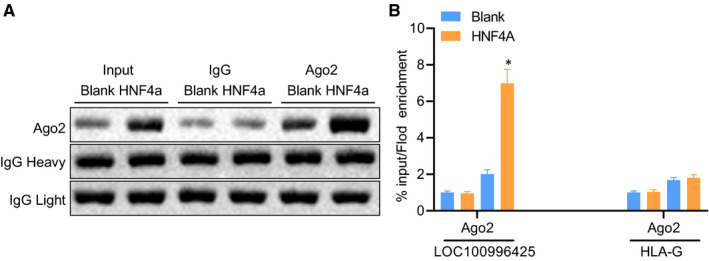
LOC100996425 directly regulates HNF4A based on RNP‐IP analysis. A, Ago‐2 immunoprecipitation revealed by immunoblotting after RNP‐IP. B, enriched LOC100996425 mRNA expression in the Ago‐2 immunoprecipitation complex, verified by RT‐qPCR. The enrichment of LOC100996425 mRNA expression was observed in the co‐immunoprecipitation RNA complex in the Ago‐2 loaded with HNF4A (HLA‐G mRNA serving as a negative control). The data are presented as mean ± standard deviation from three independent experiments and analysed by unpaired *t* test. RNP‐IP, ribonucleoprotein immunoprecipitation; IgG, immunoglobulin G. *P < .05 vs. the RNA in the normal IgG co‐immunoprecipitation

RNP‐IP was employed to further evaluate the physical interaction between HNF4A and LOC100996425 in DU‐145 cells. In accordance with the Western blot analysis, the rat anti‐human Ago‐2 monoclonal antibody was employed for the identification of the precipitated Ago‐2 (Figure 5A). In the immunoprecipitation complex with Ago‐2 comprised of HNF4A and LOC100996425, enrichment of LOC100996425 mRNA in the Ago‐2 immunoprecipitation RNA complex of overexpression of HNF4A was about 6.99 times that of the controls (Figure 5B). The human leukocyte antigen‐G (HLA‐G) mRNA was selected as NC for RNP‐IP analysis owing to its lack of HNF4A binding sites in 3'UTR.^25^ The enrichment level of HLA‐G mRNA among any of the sample groups did not exhibit significant change (Figure 5B). In summary, HNF4A binding to LOC100996425 in Ago‐2/RISC.

### 3.5 Inhibition of LOC100996425 or overexpression of HNF4A suppresses PCa cell proliferation

**FIGURE 6:**
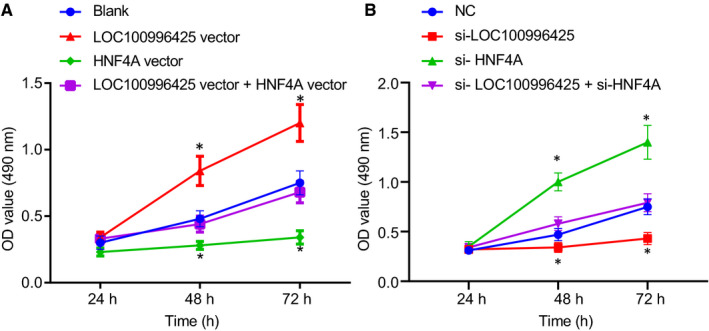
Inhibition of LOC100996425 or overexpression of HNF4A leads to the suppression of PCa cell proliferation detected by MTT assay. A, cell viability at corresponding time‐points detected using MTT assay after overexpression of LOC100996425 and/or HNF4A in DU‐145 cells; B, cell viability at corresponding time‐points detected using MTT assay after silencing of LOC100996425 and/or HNF4A in DU‐145 cells. OD, optical density; MTT, 3‐(4,5‐dimethyl‐2‐thiazolyl)‐2,5‐diphenyl‐2‐H‐tetrazolium bromide. The data were expressed as mean ± standard deviation, and two‐way ANOVA was used to compare the data among groups at different time‐points, followed by Bonferroni post hoc test. *, vs. the blank or NC groups, *P* < .05

The effects of LOC100996425 and HNF4A on PCa cell proliferation were investigated using an MTT assay. As depicted in Figure 6A, B, cell viability increased in the cells transfected with overexpressed LOC100996425 or silenced HNF4A plasmids and reduced in the cells transfected with silenced LOC100996425 or overexpressed HNF4A plasmids in comparison with cells without transfection or cells transfected with NC. There was no remarkable difference observed in terms of cell viability between the cells co‐transfected with overexpressed LOC100996425 vector and overexpressed HNF4A plasmids and cells co‐transfected with silenced LOC100996425 and silenced HNF4A plasmids.

### 3.6 Inhibition of LOC100996425 suppresses PCa cell migration by up‐regulating HNF4A

**FIGURE 7:**
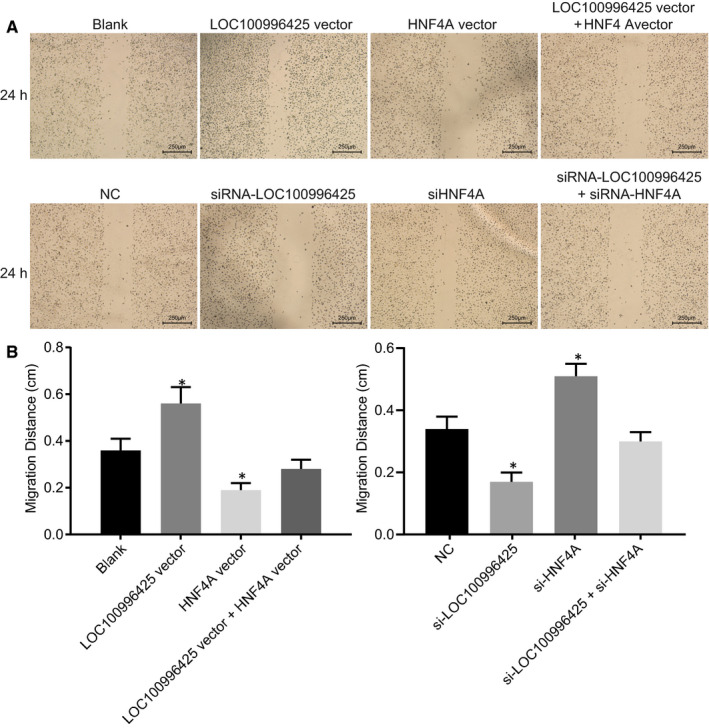
Inhibition of LOC100996425 or overexpression of HNF4A suppresses PCa cell migration. A, images showing cell migration scratch by Scratch test; B, cell migration distance after inhibition of LOC100996425 or overexpression of HNF4A, the results were obtained from three independent experiments. The data were expressed as mean ± standard deviation and analysed by the one‐way ANOVA, followed by Tukey's post hoc test. *P < .05 vs. the cells without transfection or cells transfected with NC

The effects of LOC100996425 and HNF4A on PCa cell migration were assessed by scratch test (Figure 7A, B). Compared with the cells without transfection or cells transfected with NC, the cell migration increased by LOC100996425 overexpression or HNF4A silencing, while reduced by LOC100996425 silencing or HNF4A overexpression. There was no significant difference exhibited in relation to cell migration when overexpressing both LOC100996425 and HNF4A or silencing both LOC100996425 and HNF4A.

### 3.7 Inhibition of LOC100996425 inhibits PCa cell cycle entry and promotes cell apoptosis through overexpression of HNF4A

**FIGURE 8:**
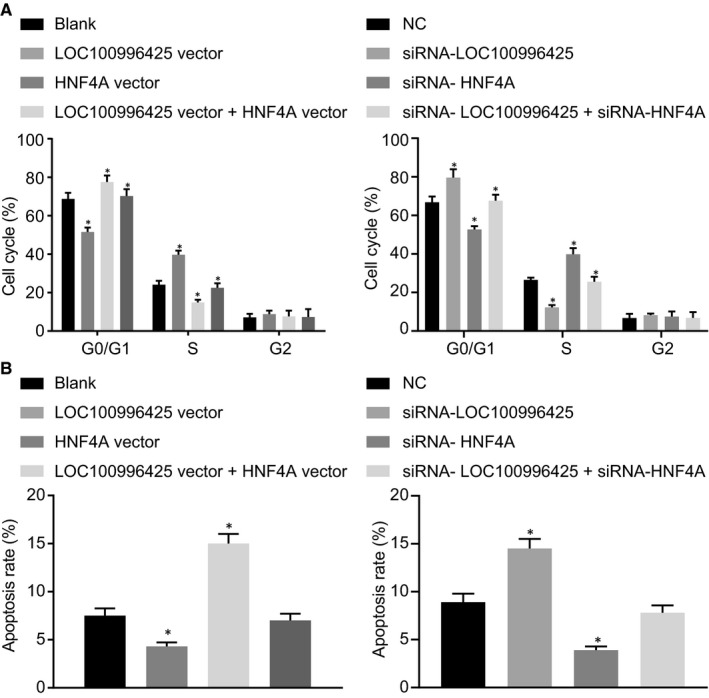
Inhibition of LOC100996425 or overexpression of HNF4A inhibits cell cycle and promotes PCa cell apoptosis. A, cell cycle distribution detected by flow cytometry. B, cell apoptosis detected by Annexin‐V/PI staining. The data were expressed as mean ± standard deviation and analysed by the one‐way ANOVA, followed by Tukey's post hoc test. *P < .05 vs. the cells without transfection or cells transfected with NC

As illustrated in Figure 8A, B, compared with the cells without transfection or cells transfected with NC, both the G1 phase cells and cell apoptosis rate reduced in the cells transfected with either overexpressed LOC100996425 or silenced HNF4A plasmids with an increased number of S phase cells; G1 phase cells and apoptosis rate increased in the cells transfected with silenced LOC100996425 or overexpressed HNF4A plasmids with decreased S phase cells. There was no marked difference in cell cycle distribution and apoptosis rate when overexpressing both LOC100996425 and HNF4A or silencing both LOC100996425 and HNF4A.

### 3.8 Inhibition of LOC100996425 up‐regulates HNF4A to inactivate the AMPK/mTOR pathway

**FIGURE 9:**
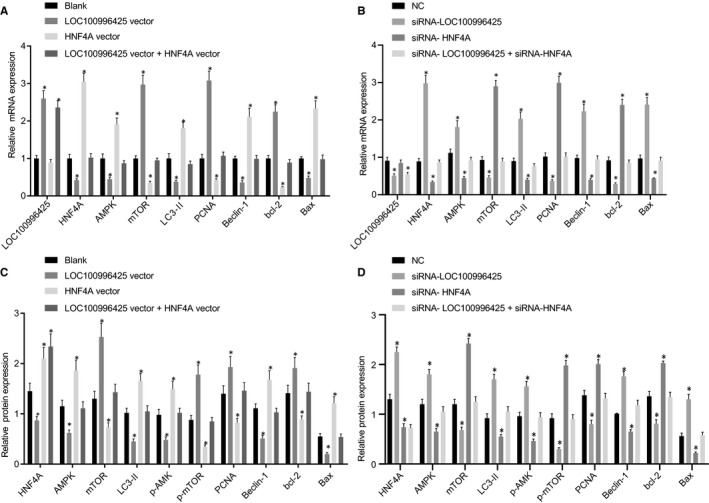
Inhibition of LOC100996425 results in the up‐regulation of HNF4A and inactivation of the AMPK/mTOR pathway. A, relative mRNA expressions of LOC100996425, HNF4A, AMPK, mTOR, LC3‐II, Beclin‐1, Bcl‐2, Bax and PCNA after treatment with overexpression of LOC100996425 and/or HNF4A in DU‐145 cells as determined by RT‐qPCR. B, relative protein expressions of LOC100996425, HNF4A, AMPK, mTOR, LC3‐II, Beclin‐1, Bcl‐2, Bax and PCNA after treatment with overexpression of LOC100996425 and/or HNF4A in DU‐145 cells as determined by Western blot analysis. C, relative mRNA expressions of HNF4A, AMPK, mTOR, LC3‐II, Beclin‐1, Bcl‐2, Bax and PCNA after treatment with silencing of LOC100996425 and/or HNF4A in DU‐145 cells as determined by RT‐qPCR. D, relative protein expressions of HNF4A, AMPK, mTOR, LC3‐II, Beclin‐1, Bcl‐2, Bax and PCNA after treatment with silencing of LOC100996425 and/or HNF4A in DU‐145 cells as determined by Western blot analysis. The data were expressed as mean ± standard deviation and analysed by the one‐way ANOVA, followed by Tukey's post hoc test. AMPK, adenosine 5’‐monophosphate‐activated protein kinase; mTOR, mammalian target of rapamycin. *P < .05 vs. the cells without transfection or cells transfected with NC

Compared to the cells without transfection or cells transfected with NC, overexpression of LOC100996425 reduced the mRNA and protein levels of HNF4A, AMPK, LC3‐II, Beclin‐1 and Bax, as well as the protein expression of p‐AMPK phosphorylation, but increased the mRNA and protein levels of mTOR, Bcl‐2 and PCNA as well as the protein expression of p‐mTOR; further, LOC100996425 and HNF4A co‐overexpression recovered the expression of HNF4A, AMPK, LC3‐II, Beclin‐1, Bax and p‐AMPK reduced by LOC100996425 overexpression, and decreased the level of mTOR, Bcl‐2, PCNA and p‐mTOR induced by LOC100996425 overexpression (Figure 9A‐C). While silencing LOC100996425 induced the opposite phenomenon, moreover, silencing LOC100996425 and HNF4A decreased the expression of HNF4A, AMPK, LC3‐II, Beclin‐1 Bax and p‐AMPK increased by silencing LOC100996425 and recovered the level of mTOR, Bcl‐2, PCNA and p‐mTOR reduced by silencing LOC100996425 (Figure 9B‐D).

**FIGURE 10:**
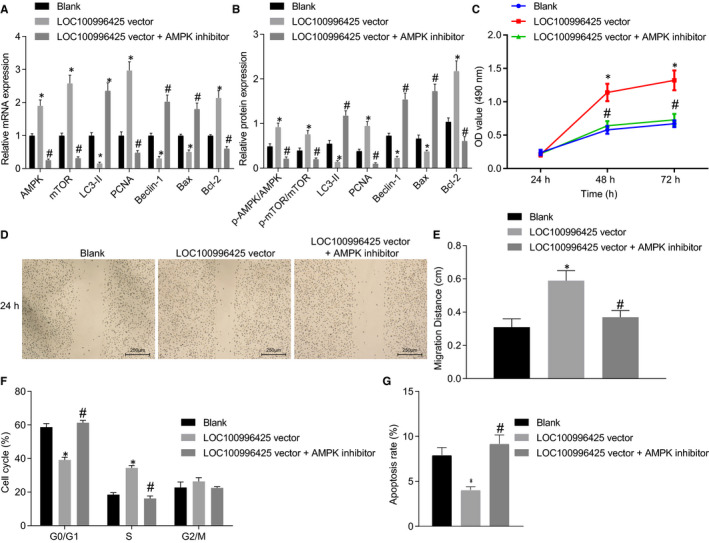
LOC100996425 affects proliferation, migration and apoptosis of PCa cells through the AMPK/mTOR pathway. A, mRNA expression of AMPK, mTOR, LC3‐II, Beclin‐1, Bcl‐2, Bax and PCNA determined by RT‐qPCR. B, protein expression of AMPK, mTOR, LC3‐II, Beclin‐1, Bcl‐2, Bax and PCNA and phosphorylation of AMPK and mTOR. C, protein expression histogram, the results were obtained from three independent experiments. D, cell viability assessed by MTT assay. E, cell migration assessed by scratch test. F, cell cycle distribution assessed by flow cytometry. G, cell apoptosis assessed by flow cytometry. The data were expressed as mean ± standard deviation, and two‐way ANOVA was used to compare the data among groups at different time‐points, followed by Bonferroni post hoc test. And data among groups were analysed by the one‐way ANOVA, followed by Tukey's post hoc test. *P < .05 vs. the cells without transfection

As depicted in Figure 10A‐C, AMPK inhibitor reversed decreased expression of LC3‐II, Beclin‐1 and Bax as well as the elevated PCNA, Bcl‐2 mRNA and protein expression caused by LOC100996425 overexpression. Further, AMPK inhibitor reversed the elevated cell proliferation ability (Figure 10D), migration ability (Figure 10E) and cell cycle entry (Figure 10F), as well as the reduced rate of apoptosis (Figure 10G) induced by LOC100996425 overexpression.

The expression of LOC100996425 in different PCa cells and normal prostate cells WPMV‐1 was detected by RT‐qPCR. The results showed that compared with WPMV‐1, the mRNA expression of LOC100996425 in PC‐3, 22RV1 and DU‐145 cells was significantly higher, and expression of LOC100996425 in DU‐145 cells was the most obvious (Figure S2), while that in LNCap cells decreased.

## 4 DISCUSSION

LncRNAs represent a significant proportion of the non‐coding transcripts in the human genome, which are evolutionally conserved and biologically functional.^26^ HNF4A was also correlated with the pathogenesis of different cancers,^27^, ^28^ and it was known that HNF4A was down‐regulated by the AMPK energy‐sensing kinase.^29^ However, correlation of LOC100996425, HNF4A and AMPK/mTOR pathway was still to be elucidated. Hence, our objective was exploring the mechanisms by which LOC100996425 targets HNF4A and the processes of cell proliferation, migration, apoptosis and autophagy in human PCa through the AMPK/mTOR pathway.

Initially, PCa tissues were observed to have exhibited high expression of LOC100996425 while poor expression of HNF4A was detected in comparison with the adjacent normal tissues. Multiple lncRNAs have been reported to regulate specific gene loci by recruiting and binding to PRC2 protein complexes, with literature suggesting that PRC2‐mediated epigenetic modulation plays a role in tumour development.^30^ A previous study indicated that PCa risk‐related loci were enriched in lncRNA intervals.^26^ HNF4A represents a transcription factor that belongs to the nuclear hormone receptor superfamily which is predominately expressed in the liver, kidney and pancreatic islets.^31^ It has suggested that HNF4A could be a direct regulator of gene expressions through its interaction with gene transcriptional regulatory elements.^32^ In addition, the potential function of HNF4A from a clinical perspective has been highlighted in previous studies, which has highlighted HNF4A as a potential and effective diagnostic tool in the process of elucidating PCa primary metastasis.^18^ Evidence has been presented suggesting the existence of reciprocal binding sites between HNF1B and HNF4A within the regulatory regions of each factor, which sanction the regulatory relationship between HNF1B and HNF4A.^32^ The overall survival rate of patients with lower expression of LOC100996425 was found to be significantly higher than those with high expression, based on the Kaplan‐Meier survival curve results. Furthermore, various lncRNAs have been earmarked due to their ability to predict the survival and prognoses of PCa patients: the overexpression of lncRNA PVT1 suggested a poor overall survival and disease‐free survival of PCa patients and lncRNA ATB was demonstrated to have predictive capabilities in PCa patients with a poor prognosis.^33^, ^34^

HNF4A was subsequently predicted to be a target gene of LOC100996425 based on the target prediction programme and luciferase activity determination, with the RNP‐IP results providing further verification indicating that LOC100996425 regulates HNF4A from a targeted manner. Following transfection, elevated expressions of AMPK, LC3‐II, Beclin‐1, Bax and AMPK phosphorylation in the siLOC100996425 and HNF4A vector groups were observed, while diminished expressions of mTOR, Bcl‐2 and PCNA, with the LOC100996425 and siHNF4A groups exhibiting contrasting trends. It has been suggested that HNF4A expression could be modulated by metformin through the AMPK pathway.^29^ It has been shown that metformin combined with 2‐deoxyglucose participated in autophagy and apoptosis in PCa cells with the involvement of AMPK pathway.^35^ Autophagy‐related proteins (LC3 and Beclin‐1) were remarkably associated with lymph node, hepatic metastasis and vessel invasion, all of which culminate in the poor survival of patients with PCa.^36^, ^37^ The imbalance of Bcl‐2/Bax largely triggered by the undue expression of Bcl‐2, and expression of PCNA affects cell proliferation and apoptosis rates in PCa.^38^, ^39^ AMPK plays a functional role in the regulation of energy homeostasis and performs as a metabolic hub, which regulates adenosine triphosphate concentrations.^40^, ^41^ AMPK regulates metabolism in reaction to energy demand by responding to AMP alterations.^42^ Studies have suggested that AMPK can inhibit mTOR signalling by phosphorylating TSC2, which is an upstream regulator of mTOR.^43^ Moreover, reports have suggested that mTOR could act as an ATP sensor in eukaryotic cells through the direct modulation of its activity via the alteration of ATP concentration,^42^ which may well elucidate the mechanism by which LOC10099642 overexpression activates the AMPK pathway. During the present study, we verified that LOC100996425 influenced proliferation, migration and apoptosis of PCa cells through AMPK/mTOR pathway. mTOR is a typical protein that plays an important role in regulating cell growth and proliferation, differentiation, migration and survival, including mTOR Complex 1 (mTORC1) and mTOR Complex 2.^41^ AMPK plays the role of a direct activator helping to regulate cellular growth and metabolic control, ultimately validating its ideal therapeutic role in PCa through the elevation of lipogenesis and activation of the mTORC1 pathway.^44^ Autophagy in PCa cells after drug treatment has been reported to be independent of AMPK activation.^45^ HNF4A is regulated by AMPKα signalling and resides upstream of WNT signalling via WNT5A, whose knockdown activity results in cell cycle arrest, cyclin down‐regulation and tumour growth inhibition,^29^ findings of which were all consistent the results of the current study. A previous study reported that vitamin D₃ combined with metformin exhibits synergistic effects on cell proliferation and apoptosis, underlying mechanisms associated with G1/S cell cycle arrest, activation of p‐AMPK accompanied by the suppression of downstream mTOR signalling, as well as reduced c‐Myc expression and p‐Bcl‐2 protein level.^46^ Additionally, the inactivation of mTOR pathway has been previously found to promote autophagic cell death in PCa cells, which was also observed in our study.^47^

## 5 CONCLUSION

In conclusion, LOC100996425 knockdown by siRNA and promotion of HNF4A expression leads to inhibition of cell proliferation, migration and promotion of apoptosis in human PCa, which aids in the prevention of the deterioration effects of PCa, associated with the mechanisms linked with the AMPK/mTOR pathway. Our study provides evidence highlighting the therapeutic potential of lncRNAs, and their capabilities as promising prognostic markers. However, additional studies with larger sample size are required for further verification of the aforementioned findings.

## CONFLICT OF INTEREST

The authors declare that they have no competing interests.

## AUTHOR CONTRIBUTIONS

**Xiuyan Wang:** Conceptualization (equal); Formal analysis (equal); Methodology (equal). **Bin Song:** Data curation (equal). **Mingcui Zang:** Writing‐original draft (equal). **He Ji:** Investigation (equal); Visualization (equal). **He Yang:** Supervision (equal). **Shuang Jiang:** Software (equal); Validation (equal). **Xiao Yang:** Conceptualization (equal); Writing‐review & editing (equal).

## Supporting information

Fig S1

Fig S2

Tables S1‐2

Table S3

## Data Availability

Research data are not shared.
