# Supplementary figures and images for: LOC100996425 acts as a promoter in prostate cancer by mediating hepatocyte nuclear factor 4A and the AMPK/mTOR pathway
Source: J Cell Mol Med. 2021 Jul 26;25(17):8174–86. doi: 10.1111/jcmm.16657 (PMC8419185; doi:10.1111/jcmm.16657)

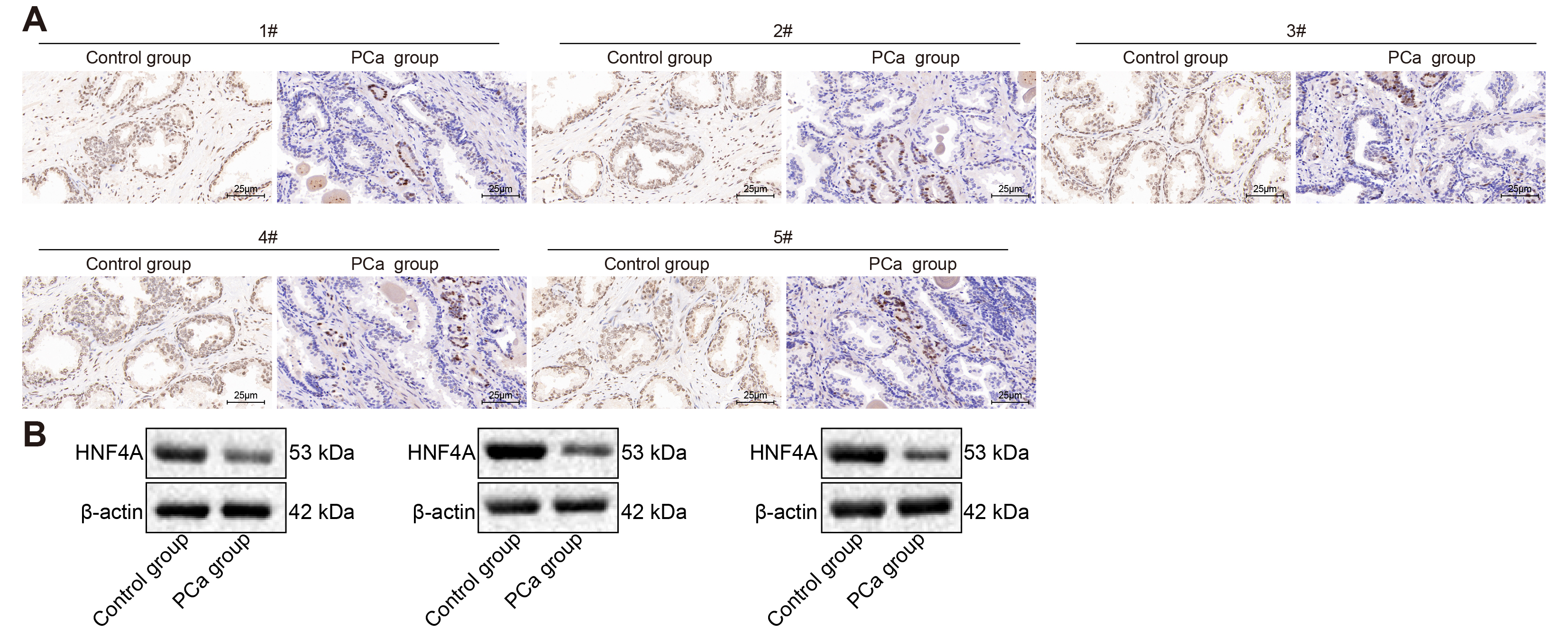

Supplement: Supplementary file 1 — Fig S1 [file JCMM-25-8174-s003.jpg]

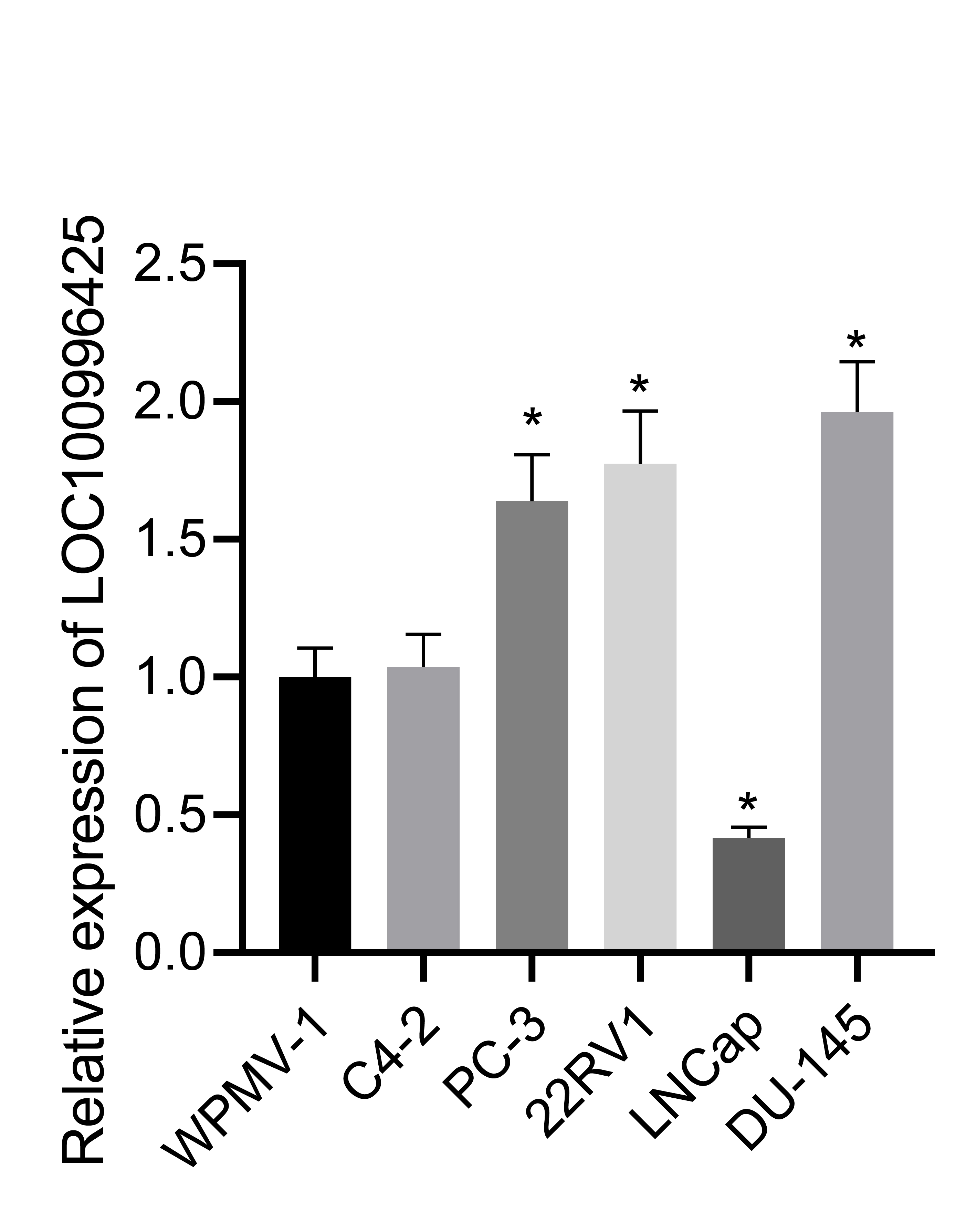

Supplement: Supplementary file 2 — Fig S2 [file JCMM-25-8174-s004.jpg]
