# Supplementary material for: LOC100996425 acts as a promoter in prostate cancer by mediating hepatocyte nuclear factor 4A and the AMPK/mTOR pathway
Source: J Cell Mol Med. 2021 Jul 26;25(17):8174–86. doi: 10.1111/jcmm.16657 (PMC8419185; doi:10.1111/jcmm.16657)
Supplement: Supplementary file 3 — Tables S1‐2 [file JCMM-25-8174-s001.docx]

**Table S1.** siRNA sequences of related genes

| Gene | Primer sequence |
| --- | --- |
| si-LOC100996425-1 | GCTTCCCTCTAGCGTCATA |
| si-LOC100996425-2 | CCTTTGAAACTTAGCCATT |
| si-LOC100996425-3 | GCAAGGGAATCAGGTTTAA |
| si-HNF4A-1 | GCTGATAGCGAAACGGATA |
| si-HNF4A-2 | GCTCCAGTGGAGAGTTCTT |
| si-HNF4A-3 | GCTGTCATGTCTGCACTTA |
| si-NC | GCTTAGCGAAACGTGACTA |

Note: LOC100996425, long noncoding RNA LOC100996425; HNF4A, hepatocyte nuclear factor 4A; NC, negative control.

**Table S2.** Primer sequences of related genes for reverse transcription-quantitative polymerase chain reaction

| Gene | Primer sequence |
| --- | --- |
| LOC100996425 | F: 5′-CTTCCCGCAGAGCTAAACCA-3' |
|  | R: 5′-TCACCTCCTTCCCACTGCTA-3' |
| HNF4A | F: 5′-GAGGAACCAGTGCCGCTACT-3' |
|  | R: 5′-TCTGGACGGCTTCCTTCTTC-3' |
| AMPK | F: 5′-GGGTGAAGATCGGACACTACGT-3' |
|  | R: 5′-TTGATGTTCAATCTTCACTTTG-3' |
| mTOR | F: 5'-CTGGGACTCAAATGTGTGCAGTTC-3' |
|  | R: 5'-GAACAATAGGGTGAATGATCCGGG-3' |
| Bcl-2 | F: 5'-ATGTGTGTGGAGAGCGTCAACC-3' |
|  | R: 5'-TGAGCAGAGTCTTCAGAGACAGCC-3' |
| LC3 | F: 5'-CCACACCCAAAGTCCTCACT-3' |
|  | R: 5'-CACTGCTGCTTTCCGTAACA-3' |
| Beclin-1 | F: 5'-TGTCACCATCCAGGAACTCA-3' |
|  | R: 5'-CTGTTGGCACTTTCTGTGGA-3' |
| Bax | F: 5'-ATGAAGACAGGGGCCCTTT-3' |
|  | R: 5'-ATGGTGAGTGAGGCGGTGA-3' |
| PCNA | F: 5'-CGGTTACTGAGGGCGAGAAG-3' |
|  | R: 5'-GCTGAGACTTGCGTAAGGGA-3' |
| GAPDH | F: 5'-GACAGTCAGCCGCATCTTCT-3' |
|  | R: 5'-GCGCCCAATACGACCAAATC-3' |

Note: HNF4A, hepatocyte nuclear factor 4A; AMPK, adenosine 5’-monophosphate-activated protein kinase; mTOR, mammalian target of rapamycin; Bcl-2, B-cell lymphoma-2; LC3, light chain 3; PCNA, proliferating cell nuclear antigen; F, forward; R, reverse.
